# LncRNA LINC00963 promotes colorectal cancer cell proliferation and metastasis by regulating miR-1281 and TRIM65

**DOI:** 10.3892/mmr.2021.12421

**Published:** 2021-09-07

**Authors:** Haidong Lv, Dixia Zhou, Guoqing Liu

**Affiliations:** Department of Tumor Surgery, Qinghai Provincial People's Hospital, Xining, Qinghai 810007, P.R. China

**Keywords:** LINC00963, microRNA-1281, tripartite motif-containing 65, colorectal cancer

## Abstract

Reportedly, long-chain non-coding RNA LINC00963 features prominently in cancer biology. However, functional details of LINC00963 in colorectal cancer (CRC) remain to be elucidated. Reverse transcription-quantitative (RT-q)PCR was performed to examine LINC00963 and microRNA (miR)-1281 expression levels in 53 matched pairs of cancerous and non-cancerous tissues from patients with CRC. Tripartite motif-containing 65 (TRIM65) protein expression in CRC cells was detected via western blot analysis. Furthermore, LINC00963 overexpression plasmid, LINC00963 small interfering RNA, miR-1281 mimics or miR-1281 inhibitors were transfected into CRC cells, and Cell Counting Kit-8, colony formation and Transwell assays were adopted to study the effects of LINC00963 and miR-1281 on the malignant phenotypes of CRC cells. Bioinformatics analysis, dual-luciferase, RNA pull-down and immunoprecipitation assays, RT-qPCR and western blot analysis were performed to investigate the regulatory relationship between LINC00963, miR-1281 and TRIM65. LINC00963 was highly expressed in CRC tissues and cells, while miR-1281 was downregulated. Functionally, LINC00963 facilitated the proliferation, colony formation, migration and invasion of CRC cells, and increased the expression levels of Ki67, matrix metalloproteinase (MMP)2 and MMP9, while miR-1281 had the opposite biological functions. Mechanistically, LINC00963 sponged miR-1281 and repressed its expression in CRC cells, resulting in the upregulation of TRIM65. LINC00963 positively regulates TRIM65 in CRC progression by repressing miR-1281 expression, showing potential as a therapeutic target for treating CRC.

## Introduction

Colorectal cancer (CRC) is a common gastrointestinal cancer accounting for ~6.1% of all cancer cases (1). For patients with distant metastasis and recurrent disease, the prognosis is poor (2). It is very important to clarify the mechanism underlying CRC progression and identify novel targets for the treatment of CRC.

It is reported that CRC is a heterogeneous disease, and its occurrence and development is a multi-step process, accompanied by complicated changes at the molecular level (3). Long non-coding RNAs (lncRNAs) are incapable of encoding proteins and participate in regulating various biological processes (4–8). For instance, lnc-GNAT1-1 is downregulated in CRC, and it serves a tumor-suppressive role by regulating Raf kinase inhibitor protein/NF-κB/Snail signaling (6). Moreover, lncRNA SNHG15 is highly expressed in CRC tissues, which is associated with lymph node and liver metastases (7,8). Notably, LINC00963 serves an important role in regulating the proliferation and migration of CRC cells (9). However, the expression pattern, biological functions and underlying mechanism of LINC00963 in CRC have not been fully elucidated.

MicroRNAs (miRNAs/miRs) are non-coding small RNAs of 19–23 nucleotides in length, and are another class of regulatory factors in CRC progression (10). In eukaryotes, miRNAs modulate the expressions of >30% of genes (11,12). Numerous miRNAs are dysregulated in CRC, such as miR-533, miR-205, miR-203a-3p, miR-127-3p and miR-18a-5p, and these miRNAs promote or repress the malignant biological behaviors of CRC cells (13–16). miR-1281 is reported to function as a tumor suppressor in multiple cancer types (17,18), and notably, miR-1281 expression is significantly downregulated in serum exosomes of patients with CRC (19); however, the function and mechanism of miR-1281 in CRC warrant further investigation.

The present study aimed to investigate the expression pattern, biological functions and mechanism of LINC00963 in CRC progression. It was revealed that LINC00963 was highly expressed in CRC, and it promoted the malignant phenotypes of CRC cells by repressing miR-1281 expression and upregulating tripartite motif-containing 65 (TRIM65) expression.

## Materials and methods

### 

#### Tissue collection

Tissue samples collected from patients with CRC between 19 and 76 years old, including 27 females and 26 males, from Qinghai Provincial People's Hospital (Qinghai, China) between May 2016 and June 2019. After histological diagnosis, only the patients who did not receive radiotherapy or chemotherapy prior to surgery were enrolled. Pathological diagnoses of CRC were determined by three pathologists according to the eighth edition of the Union for International Cancer Control and the American Joint Committee on Cancer tumor node metastasis classification (20,21). CRC tissues and normal tissues (3.0 cm away from the tumor margin) were collected during the surgery and, after resection, the samples were rapidly frozen in liquid nitrogen, and then stored at −80°C. The collection and use of human tissue samples were approved by the Ethics Review Board of Qinghai Provincial People's Hospital (approval no. KYLL180913), and written informed consent was provided by all patients.

#### Cell lines and transfection

Human CRC cell lines (HCT116, SW480 and LoVo cells) were purchased from the American Type Culture Collection, HT29 cells and the normal colon epithelial cell line NCM460 were purchased from China Center for Type Culture Collection. The cells were maintained in Dulbecco's modified Eagle's medium (DMEM) containing 10% fetal bovine serum (FBS; both Gibco; Thermo Fisher Scientific, Inc.), 100 U/ml penicillin and 100 µg/ml streptomycin (Sigma-Aldrich; Merck KGaA) at 37°C with 5% CO_2_. The sequence of LINC00963 was cloned into pcDNA3.1 vector (Invitrogen; Thermo Fisher Scientific, Inc.) to construct overexpression plasmids (pcDNA3.1-LINC00963, LINC00963), with empty vectors used as the controls (pcDNA3.1-vector, NC). LINC00963 small interfering (si)RNAs (si-LINC00963#1, 5′-TTGTACAGTTGGGTAAATCGAGG-3′; and si-LINC00963#2, 5′-CCAGACACTGAACTGCCTT-3′), miR-1281 mimics (5′-UCGCCUCCUCCUCUCCC-3′), miR-1281 inhibitor (5′-AGCGGAGGAGGAGAGGG-3′), and corresponding negative controls (NCs), including si-NC (5′-UUCUCCGAACGUGUCACGUTT-3′), miR-NC (5′-UCACAACCUCCUAGAAAGAGUAGA-3′) and miR-NC inhibitor (5′-GTGTAACACGTCTATACGCCA-3′), were obtained from Invitrogen (Thermo Fisher Scientific, Inc.). The transient transfection was conducted with final concentrations of 50 nM of siRNAs, miRNA mimics and inhibitor, and 2 µg of overexpression plasmid by Lipofectamine^®^ 2000 (Invitrogen; Thermo Fisher Scientific, Inc.) according to the manufacturer's instructions. Lipofectamine 2000 was added with oligonucleotide or plasmid, mixed gently and incubated for 20 min at room temperature. Subsequently, the mixture was added to cell suspension (5×10^6^ cells/ml), and then incubated at 37°C and 5% CO_2_ for 6 h. Finally, the medium was replaced with DMEM supplemented with 10% FBS. Cells were incubated at 37°C for 48 h, and were harvested for reverse transcription-quantitative (RT-q)PCR, Cell Counting Kit-8 (CCK-8), colony formation, Transwell and western blot assays.

#### RT-qPCR

Total RNA of tissues and cells was extracted using TRIzol^®^ reagent (Invitrogen; Thermo Fisher Scientific, Inc.) and then reverse transcribed to obtain cDNA with a Transcriptor First Strand cDNA Synthesis kit (Roche Diagnostics) according to the manufacturer's instructions. qPCR was performed with SYBR-Green I Master (Roche Diagnostics) on the LightCycler^®^ 480 System (Roche Diagnostics). The RT-qPCR cycling conditions were as follows: Initial denaturation at 95°C for 10 min, followed by 40 cycles of denaturation at 95°C for 15 sec and annealing/elongation at 60°C for 60 sec. The relative gene expression level of LINC00963 and TRIM65 was normalized to GAPDH, and miR-1281 expression was normalized to U6, with the fold change calculated by the 2^−ΔΔCq^ method (22). The primers were designed by BGI Genomics.

#### CCK-8 assay

Briefly, 100 µl of CRC cell suspension (containing ~2×10^3^ cells) was added to each well of a 96-well plate. Then, 10 µl of CCK-8 solution (Dojindo Molecular Technologies, Inc.) was added to each well at 0, 24, 48 and 72 h. After the cells were incubated at 37°C for 2 h, the value of optical density of each well at 450 nm was measured by a microplate reader.

#### Colony formation assay

At 48 h after transfection, CRC cells were dispersed, seeded into 6-well plates (1,000 cells/well) and cultured for 2 weeks. Then, the medium was discarded, and the colonies were washed using phosphate buffered saline (PBS), fixed with 4% paraformaldehyde for 10 min at room temperature and stained with 0.1% crystal violet for 15 min at room temperature. After the colonies were washed using PBS again and dried, the colonies were observed with a light microscope (magnification, ×200; Olympus Corporation) and photographed with a camera (magnification, ×2.5; Nikon Corporation), and the number of cell colonies (containing >50 cells) was counted manually.

#### Cell invasion and migration assays

To assess the migration and invasion of CRC cells, 4×10^4^ transfected cells in 200 µl of serum-free medium were transferred into the upper compartment of each Transwell chamber (pore size, 8 µm, Corning Life Sciences), and 500 µl of DMEM with 10% FBS was loaded into the lower compartment. After the cells were cultured for 24 h, the migrated or invaded cells were washed in PBS twice and fixed with 4% paraformaldehyde for 25 min at room temperature and stained with 0.1% crystal violet for 10 min at room temperature, successively. After that, the images were captured under a light microscope (Olympus Corporation) at ×200 magnification, and cell counting was performed manually in three randomly selected visual fields. For the invasion assay, the bottom of the Transwell chambers was coated with a layer of Matrigel (1:8; Corning Life Sciences) at 37°C for 30 min; the other procedures are the same as those for the migration assay.

#### Luciferase reporter assay

LncBase Predicted v.2 database (threshold, 0.8; http://carolina.imis.athena-innovation.gr/diana_tools/web/index.php?r=lncbasev2%2Findex-predicted) and TargetScanHuman database (version 7.2; http://www.targetscan.org/vert_72/) were used to predict the potential binding sites between LINC00963 and miR-1281, and miR-1281 and TRIM65 3′-untranslated region (3′UTR) (23,24). For validating the predicted binding sites between LINC00963 and miR-1281, and miR-1281 and TRIM65 3′UTR, pmiR-RB-REPORT™ (Guangzhou RiboBio Co., Ltd.) reporter plasmids containing the wild-type (WT) or mutant type (MUT) LINC00963 and TRIM65 3′UTR sequences with predicted binding sites for miR-1281 were constructed. Then, 293T cells (4×10^4^ cells; China Center for Type Culture Collection) were cultured in DMEM with 10% FBS and were co-transfected with the reporter plasmids (80 ng) and miR-1281 mimics or control miRNA (50 nM) using Lipofectamine^®^ 2000 (Invitrogen; Thermo Fisher Scientific, Inc.) according to the manufacturer's instructions. Next, the cell culture was continued for 48 h, before the cells in each group were collected and the luciferase activities were determined with a dual-luciferase reporter assay kit (Promega Corporation) according to the instructions provided by the manufacturer. The relative luciferase activity was normalized to Renilla luciferase activity.

#### RNA immunoprecipitation (RIP) assay

The RIP assay was performed with a Magna RIP™ RNA-Binding Protein Immunoprecipitation kit (Millipore Inc.) according to the manufacturer's instructions. In brief, RIP lysis buffer was utilized to lyse the CRC cells, and 200 µl of cell lysates were immunoprecipitated with anti-argonaute 2 (Ago2; cat. no. ab32381; 1:50) or negative control anti-immunoglobulin G (IgG; cat. no. ab190475; 1:100) antibodies (Abcam) at 4°C overnight. Next, proteinase K and DNase (Beyotime Institute of Biotechnology) were used to remove the proteins and DNA in the mixture, and then the RNA was immunoprecipitated. Following that, the total RNA was extracted using a RNeasy MinElute Cleanup kit (Qiagen China Co., Ltd.). Subsequently, RT-qPCR was performed to detect the enrichment of LINC00963 as aforementioned.

#### RNA pull-down assay

Biotinylated miR-1281 or NC probes were conjugated with streptavidin beads (Beyotime Institute of Biotechnology). The biotinylated RNA was transfected into cells using Lipofectamine^®^ 2000 (Invitrogen; Thermo Fisher Scientific, Inc.) according to the manufacturer's instructions. RNA pull-down assay was performed using a Magnetic RNA-Protein Pull-Down kit (cat. no. 20164; Thermo Fisher Scientific, Inc.). The cells transfected with probes were incubated with lysis buffer on ice, and after 10 min, the mixtures were centrifuged at 13,000 × g for 10 min at 4°C and cell debris was discarded. Subsequently, the supernatants (10 mg) were incubated with magnetic beads for 2 h at 4°C, and then RNA was purified using an RNeasy Mini kit (Qiagen GmbH) according to the manufacturer's instructions. Finally, the abundance of LINC00963 was analyzed using RT-qPCR.

#### Western blot analysis

LoVo and HTC116 cells were lysed in RIPA lysis buffer (Sigma-Aldrich; Merck KGaA). Then lysates were centrifuged at 12,000 × g for 20 min at 4°C, and the total proteins were extracted. Protein concentrations were detected by a BCA protein assay kit (Thermo Fisher Scientific, Inc.). The protein sample (60 µg/lane) in each group was separated via 8% SDS-PAGE and then transferred onto polyvinylidene fluoride membranes (Beyotime Institute of Biotechnology). After that, the membranes were blocked with 3% bovine serum albumin and then incubated overnight at 4°C with primary antibodies against TRIM65 (Aviva Systems Biology; cat. no. OAAB08057; 1:500), Ki67 (Abcam; cat. no. ab15580; 1:500), matrix metalloproteinase 2 (Abcam; cat. no. ab92536; 1:500), matrix metalloproteinase 9 (Abcam; cat. no. ab76003; 1:500) and GAPDH (Abcam; cat. no. ab8245; 1:3,000). GAPDH was the internal reference. Next, Tris-buffered saline with 0.05% Tween 20 (TBST) was utilized for rinsing the membranes 3 times for 15 min each time. Next, horseradish peroxidase-labeled goat anti-rabbit and anti-mouse IgG (H + L) (Beyotime Institute of Biotechnology; cat. nos. A0208 and A0216, respectively; 1:2,000) were used to incubate with the membranes at room temperature for 30 min. After the membranes were washed with TBST again, the protein bands were developed using an enhanced chemiluminescence (ECL) kit (Beyotime Institute of Biotechnology) and detected by ChemiDoc™ Touch Imaging System (cat. no. 1708370; Bio-Rad Laboratories, Inc.). The intensity of the bands was analyzed by ImageJ software version 1.8.0 (National Institutes of Health).

#### Statistical analysis

All experiments were performed in triplicate. Experimental data were presented as the mean ± SD. Statistical analysis was performed with SPSS 17.0 (SPSS, Inc.). Whether the data are normally distributed or not was examined by the Kolmogorov-Smirnov test. Student's unpaired t-test or one-way ANOVA (with Tukey's post-hoc test) was employed to make comparisons. Pearson's correlation analysis was used to analyze the correlation between the expressions of LINC00963 and miR-1281 in CRC tissues. The association of LINC00963 expression levels with clinicopathological characteristics was analyzed using χ^2^ test. P<0.05 was considered to indicate a statistically significant difference.

## Results

### 

#### LINC00963 expression is upregulated in CRC

**Figure 1.**
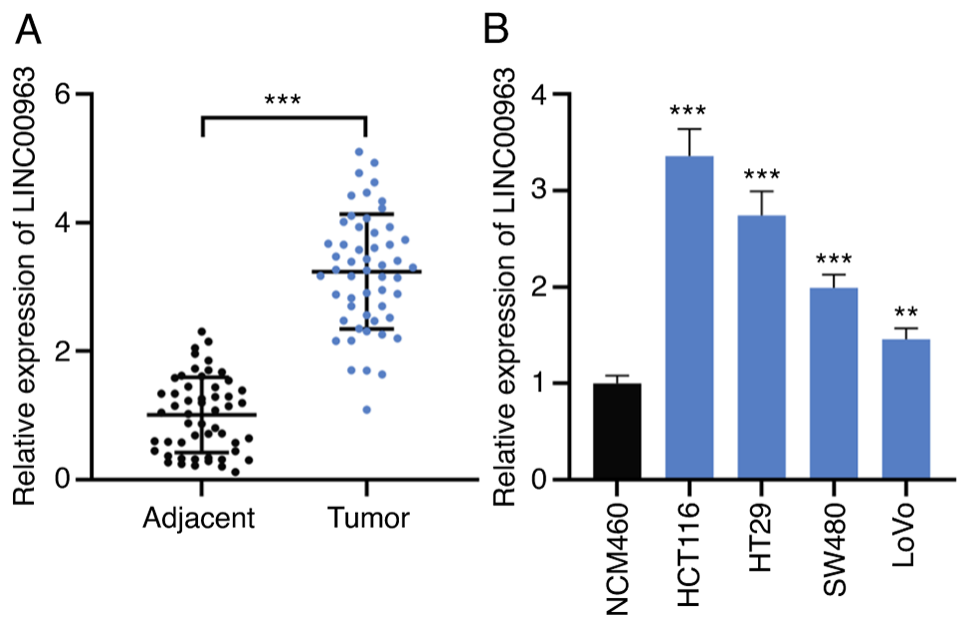
LINC00963 expression is upregulated in CRC tissues and cell lines. (A) LINC00963 expression in 53 pairs of CRC tissues and adjacent tissues was detected by RT-qPCR. (B) LINC00963 expression in normal colonic epithelial cell line (NCM460 cells) and 4 CRC cell lines (HCT116, HT29, SW480 and LoVo cells) was detected by RT-qPCR. All experiments were performed in triplicate. **P<0.01, ***P<0.001 vs. NCM460 or as indicated. CRC, colorectal cancer; RT-qPCR, reverse transcription-quantitative PCR.

Firstly, RT-qPCR was used to detect LINC00963 expressions in cancer tissues and adjacent tissues of 53 patients with CRC. As shown, LINC00963 expression in CRC was significantly upregulated, compared with that in normal tissues (Fig. 1A). Additionally, in CRC cell lines, LINC00963 expression was higher compared with in normal colonic epithelial cell line (Fig. 1B). Statistical analyses revealed that high expression of LINC00963 was significantly associated with larger tumor size (P=0.039) and lymph node metastasis (P=0.020) of the patients (Table I).

#### LINC00963 promotes proliferation, migration and invasion of CRC cells

**Figure 2.**
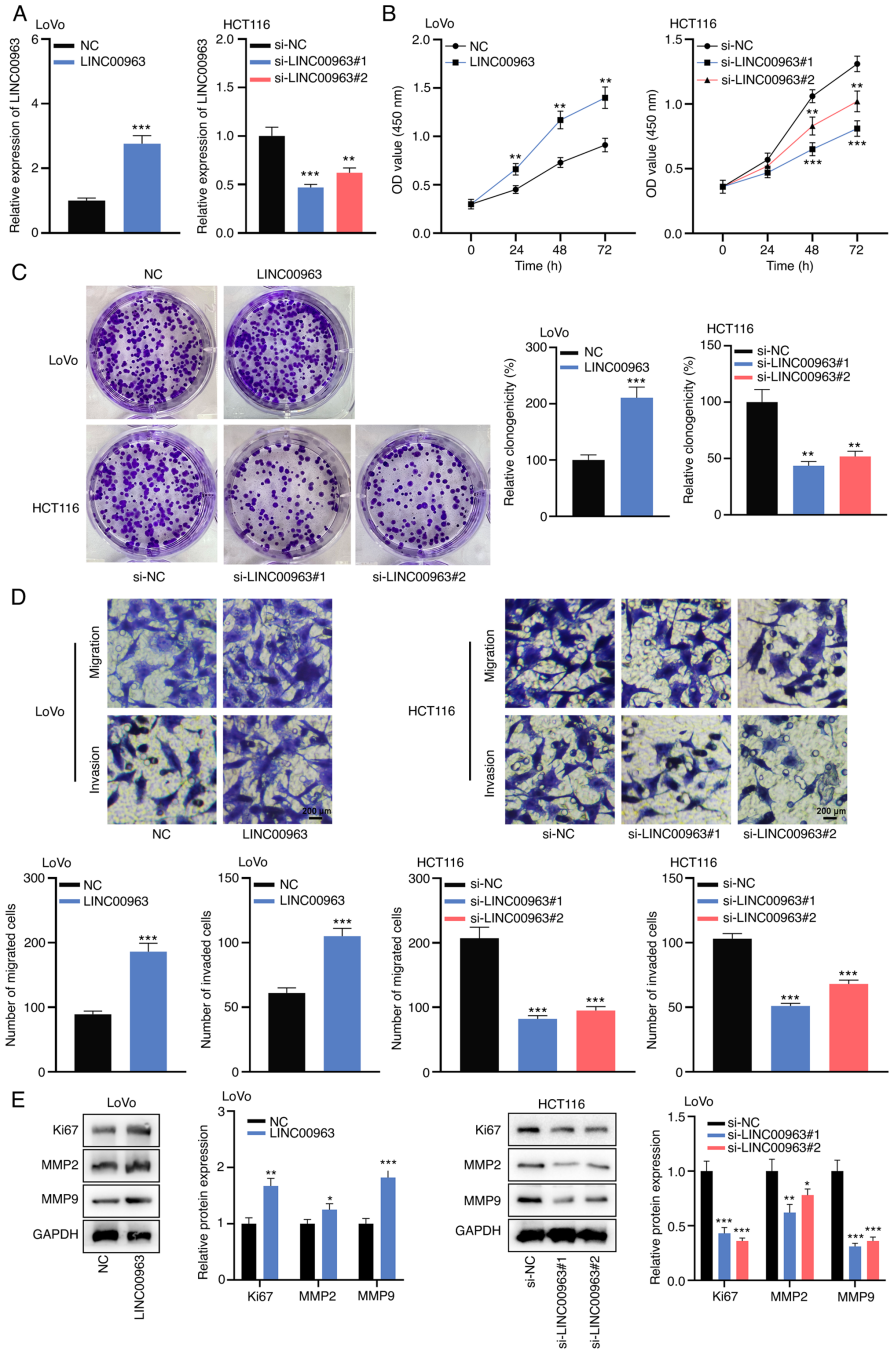
LINC00963 promotes the proliferation, migration and invasion of colorectal cancer cells. (A) LINC00963 overexpression plasmids and siRNAs were transfected into LoVo and HCT116 cells, respectively, and the relative expression of LINC00963 in LoVo and HCT116 cells after the transfection was detected by reverse transcription-quantitative PCR. (B) Cell Counting Kit-8 assay was used to detect the proliferation of LoVo cells with LINC00963 overexpression and HCT116 cells with LINC00963 knockdown. (C) Colony formation assay was used to detect the colony-forming ability of LoVo cells with LINC00963 overexpression and HCT116 cells with LINC00963 knockdown. (D) Transwell assay was used to detect the migration and invasion of LoVo cells with LINC00963 overexpression and HCT116 cells with LINC00963 knockdown. Scale bar, 200 µm. (E) Western blot assay was used to detect the expression of proliferation- and metastasis-related proteins (Ki67, MMP2 and MMP9) in LoVo cells with LINC00963 overexpression and HCT116 cells with LINC00963 knockdown. All experiments were performed in triplicate. *P<0.05, **P<0.01, ***P<0.001 vs. NC or si-NC. MMP, matrix metalloproteinase; NC, negative control; si, small interfering.

Given that among the CRC cell lines, LINC00963 had the lowest expression in LoVo cells and the highest expression in HCT116 cells, LoVo and HCT116 cells were selected as cell models for further experiments. LINC00963 overexpression plasmids were transfected into LoVo cells to construct a cell model of LINC00963 overexpression, and LINC00963 siRNAs were transfected into HCT116 cells to construct LINC00963 knockdown models. RT-qPCR confirmed that the transfection was successful (Fig. 2A). The CCK-8 assay indicated that LINC00963 overexpression significantly promoted the proliferation of LoVo cells at 24, 48 and 72 h, while knocking down LINC00963 had the opposite effects on HCT116 cells at 48 and 72 h (Fig. 2B). The colony formation assay showed that LINC00963 overexpression markedly increased the colony-forming ability of LoVo cells, but this colony-forming ability was suppressed in HCT116 cells following LINC00963 knockdown (Fig. 2C). The Transwell assays suggested that the migration and invasion of LoVo cells with LINC00963 overexpression were significantly increased, while the migration and invasion of HCT116 cells with LINC00963 knockdown were significantly decreased (Fig. 2D). Additionally, western blot analysis revealed that LINC00963 overexpression promoted the expression levels of Ki67, MMP2 and MMP9, while knockdown of LINC00963 caused the opposite effect (Fig. 2E). These results suggested that LINC00963 promoted the malignant phenotypes of CRC cells.

#### LINC00963 may function as a molecular sponge for miR-1281

**Figure 3.**
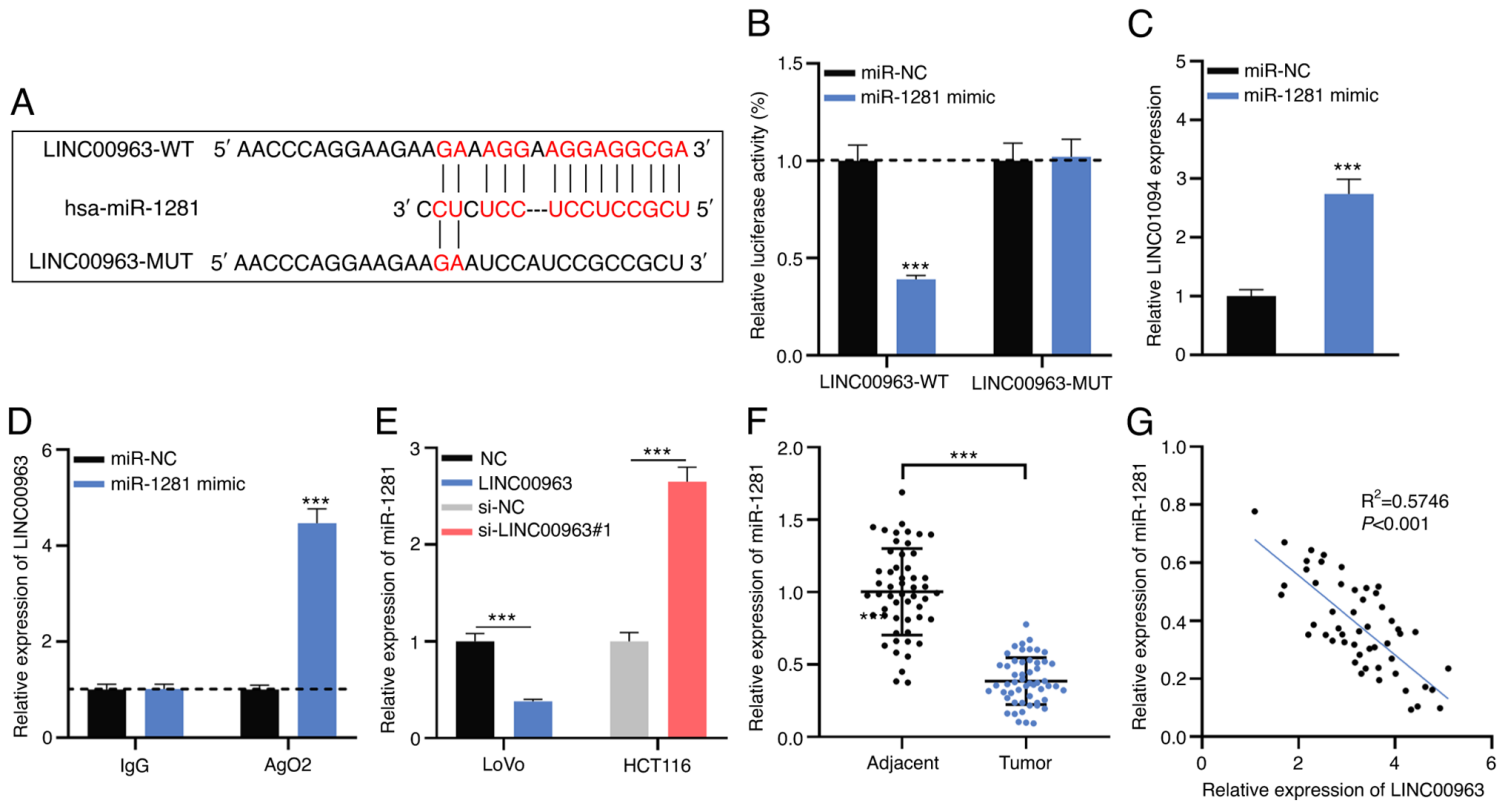
miR-1281 is a target of LINC00963 in CRC. (A) LINC00963-WT and LINC00963-MUT luciferase reporter plasmids containing the binding site for miR-1281 were constructed. (B) Targeting relationship between miR-1281 and LINC00963 was validated by a dual-luciferase reporter assay. (C) RNA pull-down and (D) RNA immunoprecipitation assays were performed to confirm that LINC00963 interacted with miR-1281 directly. (E) Expression level of miR-1281 in LoVo cells with LINC00963 overexpression and HCT116 cells with LINC00963 knockdown was detected by RT-qPCR. (F) miR-1281 expression in 53 pairs of CRC and adjacent tissues was detected by RT-qPCR. (G) The correlation between miR-1281 and LINC00963 expressions in CRC tissues was analyzed by Pearson's analysis. All experiments were performed in triplicate. ***P<0.001 vs. miR-NC. CRC, colorectal cancer; NC, negative control; miR, microRNA; WT, wild-type; MUT, mutant; Ago2, argonaute 2; si, small interfering; RT-qPCR, reverse transcription-quantitative PCR.

Using the LncBase database, it was revealed that there was a potential binding site between LINC00963 (Gene_ID: ENSG00000204054) and miR-1281 (Fig. 3A and Table SI). To verify whether LINC00963 targets miR-1281, LINC00963-WT and LINC00963-MUT luciferase reporter plasmids were co-transfected into LoVo and HCT116 cells with miR-1281 mimics or miR-NC. The results revealed that miR-1281 mimics significantly decreased the luciferase activity of the LINC00963-WT reporter plasmid but had no impact on that of LINC00963-MUT (Fig. 3B). Additionally, RNA pull-down and RIP experiments demonstrated that LINC00963 directly interacted with miR-1281 (Fig. 3C and D). Furthermore, RT-qPCR revealed that miR-1281 expression in LoVo cells with LINC00963 overexpression was significantly inhibited, and in HCT116 cells with LINC00963 knockdown, the expression of miR-1281 was markedly increased (Fig. 3E). Besides, it was found that miR-1281 expression in CRC was significantly decreased compared with in in adjacent tissues (Fig. 3F), and Pearson's correlation analysis showed that LINC00963 expression was negatively correlated with miR-1281 expression in CRC tissue (Fig. 3G). Collectively, these results implied that LINC00963 adsorbed miR-1281 and negatively regulated its expression in CRC.

#### miR-1281 represses the malignant biological behaviors of CRC cells

**Figure 4.**
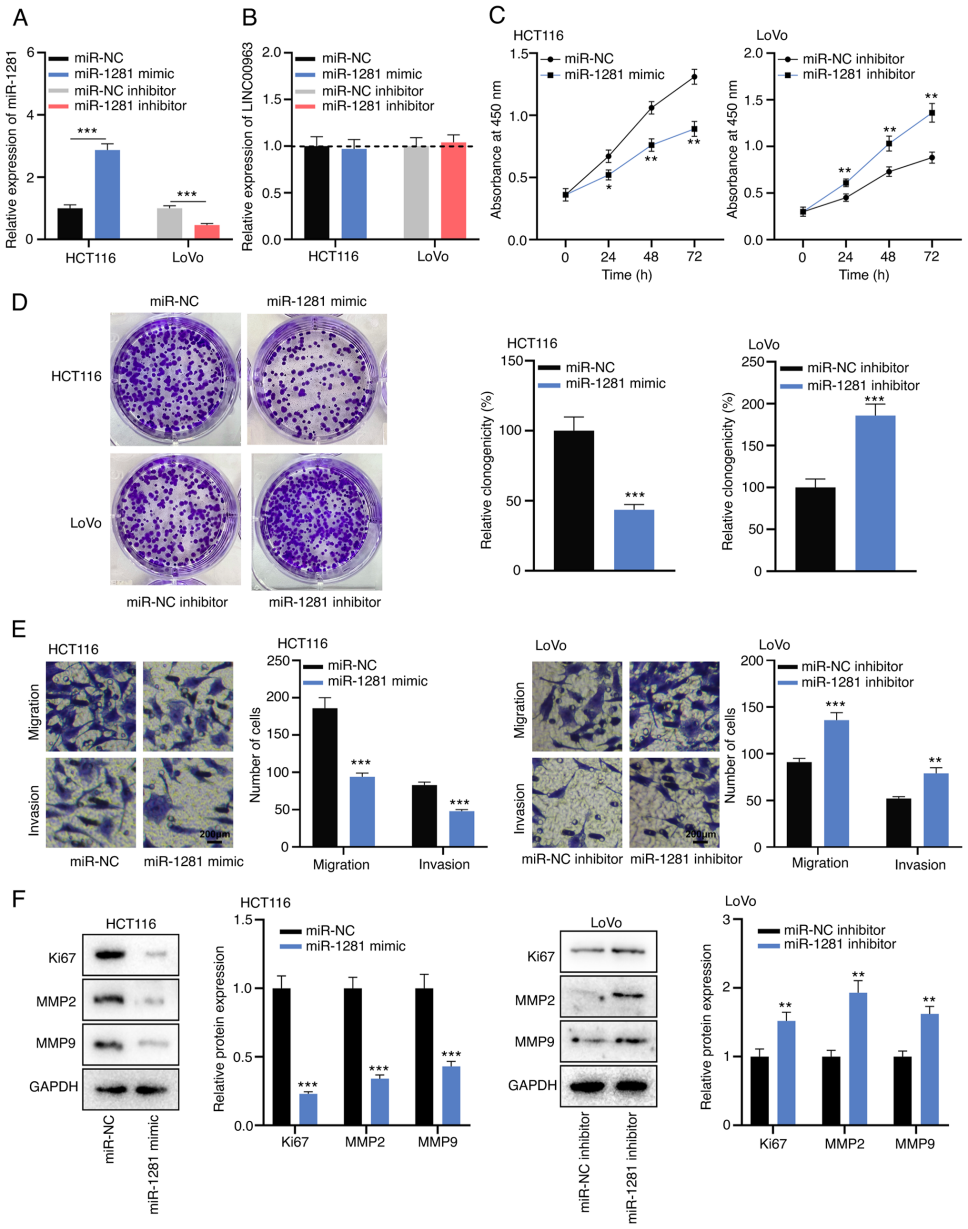
miR-1281 serves a tumor-suppressive role in colorectal cancer. (A) Expression of miR-1281 was detected by RT-qPCR in HCT116 cells transfected with miR-1281 mimics and LoVo cells transfected with miR-1281 inhibitors. (B) LINC00963 expression in HCT116 cells with miR-1281 overexpression and LoVo cells with miR-1281 inhibition was detected by RT-qPCR. (C) Cell Counting Kit-8 assay was used to detect the proliferation of HCT116 cells with miR-1281 overexpression and LoVo cells with miR-1281 knockdown. (D) Colony formation assay was used to detect the colony-forming ability of HCT116 cells with miR-1281 overexpression and LoVo cells with miR-1281 knockdown. (E) Transwell assay was used to detect the migration and invasion of HCT116 cells with miR-1281 overexpression and LoVo cells with miR-1281 knockdown. (F) Western blotting was used to detect the expression of proliferation- and metastasis-related protein (Ki67, MMP2 and MMP9) in HCT116 cells with miR-1281 overexpression and LoVo cells with miR-1281 knockdown. All experiments were performed in triplicate. *P<0.05, **P<0.01, ***P<0.001 vs. miR-NC or miR-NC inhibitor. NC, negative control; miR, microRNA; RT-qPCR, reverse transcription-quantitative PCR; MMP, matrix metalloproteinase.

To elucidate the biological function of miR-1281, miR-1281 mimics and inhibitors were transfected into HCT116 and LoVo cells, and it was confirmed that the transfection was successful via RT-qPCR (Fig. 4A). RT-qPCR also revealed that in HCT116 cells with miR-1281 overexpression and LoVo cells with miR-1281 inhibition, there was no significant change in LINC00963 expression (Fig. 4B). CCK-8, colony formation and Transwell assays revealed that overexpression of miR-1281 inhibited the proliferation, migration and invasion of HCT116 cells, while in LoVo cells, knockdown of miR-1281 had the opposite effects (Fig. 4C-E). Furthermore, western blot analysis verified that the miR-1281 mimic inhibited the expression levels of Ki67, MMP2 and MMP9, while the opposite effects were observed after transfection of miR-1281 inhibitor (Fig. 4F). These results suggested that miR-1281 inhibited the malignant biological behaviors of CRC cells.

#### TRIM65 is a target of miR-1281

**Figure 5.**
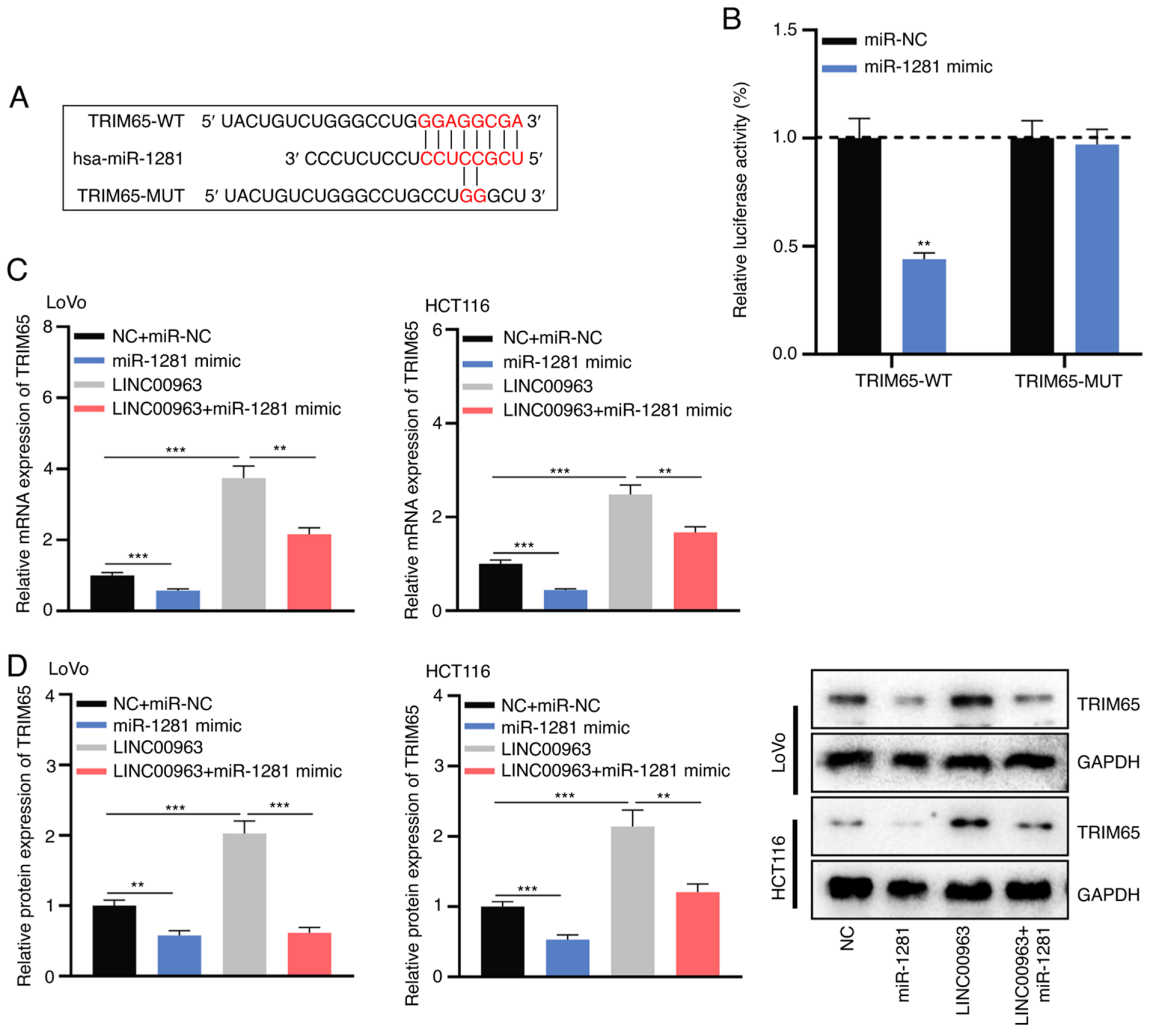
TRIM65 is a downstream target of miR-1281 in colorectal cancer. (A) TRIM65-WT and TRIM65-MUT luciferase reporter plasmids containing the binding site for miR-1281 were constructed. (B) Targeting relationship between miR-1281 and the 3′UTR of TRIM65 was verified by a dual-luciferase reporter assay. (C) LINC00963 overexpression plasmids and/or miR-1281 mimics were transfected into LoVo and HCT116 cells, and mRNA expression of TRIM65 in the transfected cells was detected by reverse transcription-quantitative PCR. (D) Protein expression of TRIM65 in the transfected cells was detected by western blot analysis. All experiments were performed in triplicate. **P<0.01, ***P<0.001 vs. miR-NC. TRIM65, tripartite motif-containing 65; MUT, mutant; WT, wild-type; miR, microRNA; UTR, untranslated region.

TargetScan predicted that there was a complementary binding site between miR-1281 and the 3′UTR of TRIM65 (Fig. 5A). To verify whether miR-1281 could bind to the 3′UTR of TRIM65, TRIM65-WT and TRIM65-MUT reporter plasmids were co-transfected into 293T cells with miR-1281 mimics or miR-NC. The results confirmed that miR-1281 mimics significantly reduced the luciferase activity of TRIM65-WT, while the luciferase activity of TRIM65-MUT was not altered (Fig. 5B). RT-qPCR and western blot analysis demonstrated that overexpression of miR-1281 significantly reduced TRIM65 expression at both the mRNA and protein level in HCT116 and LoVo cells, while LINC00963 overexpression had opposite effects; moreover, overexpression of miR-1281 partially inhibited the increase of TRIM65 expression caused by LINC00963 overexpression (Fig. 5C and D).

#### LINC00963 regulates CRC cell proliferation and metastasis via the miR-1281/TRIM65 axis

**Figure 6.**
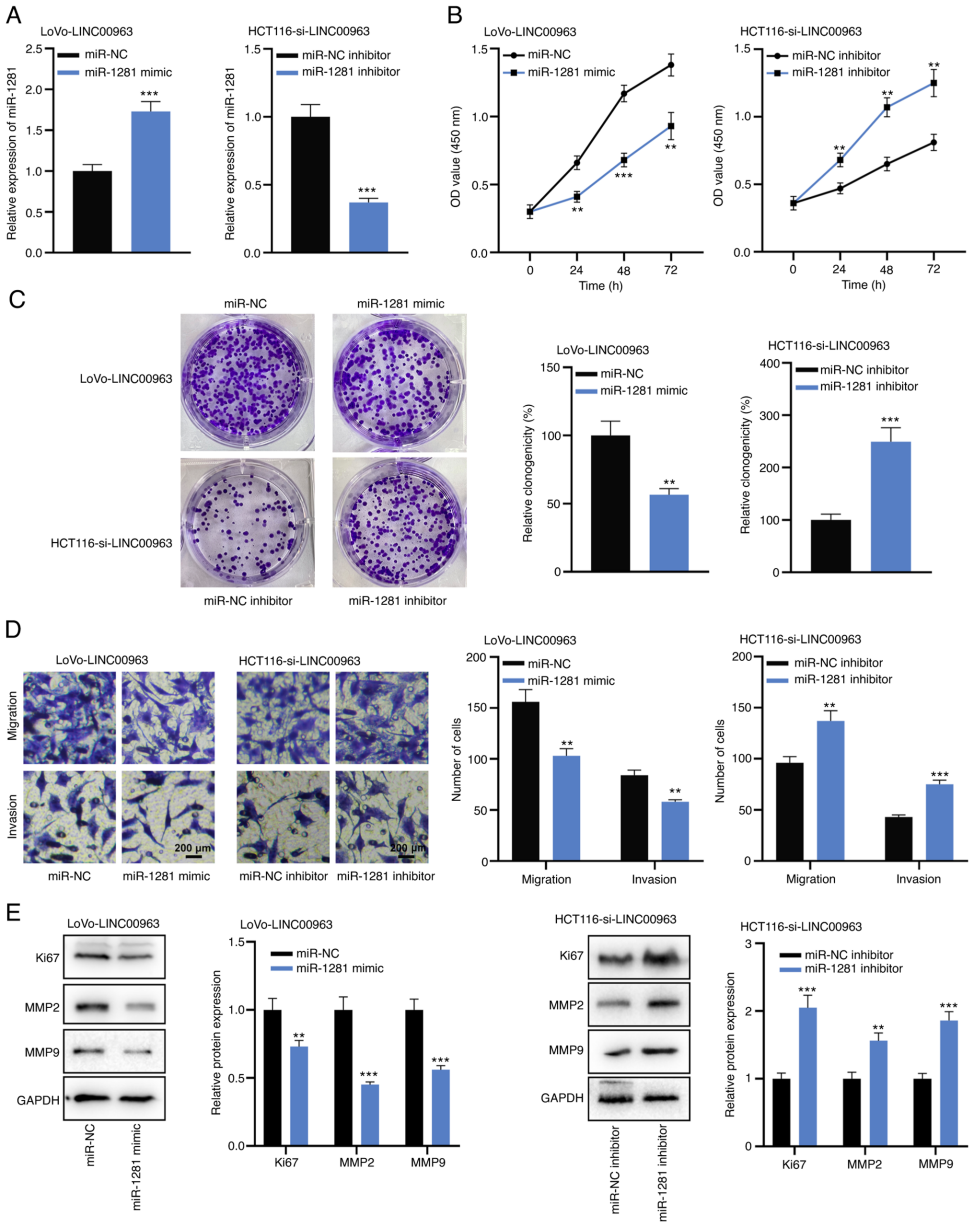
miR-1281 partially reverses the tumor-promoting effect of LINC00963 on colorectal cancer cells. (A) miR-1281 mimics were transfected into LoVo cells with LNC229094 overexpression, and miR-1281 inhibitors were transfected into HCT116 cells with LINC00963 knockdown. The expression of miR-1281 in the transfected cells was detected by reverse transcription-quantitative PCR. (B) Cell Counting Kit-8 assay was used to detect the proliferation of LoVo and HCT116 cells. (C) Colony formation assay was used to detect the colony-forming ability of LoVo cells and HCT116 cells. (D) Transwell assay was used to detect the migration and invasion capacities of LoVo and HCT116 cells. (E) Western blot assay was used to detect the expression of proliferation- and metastasis-related proteins (Ki67, MMP2 and MMP9) in LoVo cells and HCT116 cells. All experiments were performed in triplicate. **P<0.01, ***P<0.001 vs. miR-NC in LoVo cell with LINC00963 overexpression or miR-NC inhibitor in HCT116 cells with LINC00963 knockdown. NC, negative control; miR, microRNA; MMP, matrix metalloproteinase.

To clarify whether LINC00963 exerted its biological function through miR-1281, miR-1281 mimics were transfected into LoVo cells with LINC00963 overexpression, and miR-1281 inhibitors were transfected into HCT116 cells with LINC00963 knockdown; the transfection efficacy was verified via RT-qPCR (Fig. 6A). Functional experiments indicated that miR-1281 mimics could partially abrogate the effects of LINC00963 overexpression on the viability and metastasis of LoVo cells; moreover, miR-1281 inhibition partially reversed the inhibitory effect of knocking down LINC00963 on the proliferation and metastasis of HCT116 cells (Fig. 6B-E). These results supported the hypotheses that LINC00963 regulates the malignant biological behaviors of CRC cells by regulating miR-1281/TRIM65 axis.

## Discussion

The importance of lncRNAs in cancer biology has been increasingly recognized (25). Although the roles of certain lncRNAs in CRC have been reported, a large number of CRC-related lncRNAs have not been identified. LINC00963, also known as MetaLnc9, is reported to be abnormally expressed in various tumor types, and is involved in regulating the proliferation, migration and invasion of tumor cells (26–29). For example, it promotes the proliferation and invasion abilities of osteosarcoma cells by regulating the miR-204-3p/fibronectin 1 (FN1) axis (26). Its expression is upregulated in liver cancer, which promotes the proliferation of cancer cells by activating the PI3K/AKT pathway (27). In head and neck carcinomas, LINC00963 is highly expressed, and knockdown of LINC00963 reduces the ability of self-renewal, invasion and colony formation in cancer cells by regulating ABCB5 (28). LINC00963 also features prominently in the transition of prostate cancer from androgen dependence to androgen independence (29). In the present study, it was observed that LINC00963 expression was upregulated in CRC tissues and cells, which is consistent with a previous study (9); moreover, the high expression of LINC00963 was associated with larger tumor size and lymph node metastasis in patients. Functionally, LINC00963 potentiated the proliferation, colony formation, migration and invasion of CRC cells, implying that LINC00963 represents a tumor promoter in CRC, and may be a promising novel therapeutic target.

miRNAs, which are also non-coding RNAs, widely exist in human organs and tissues, and partake in the vast majority of biological processes (30). Reportedly, miR-1281 has tumor-suppressive properties in certain types of cancer; specifically, in osteosarcoma, miR-1281 expression is modulated by p53, and it is upregulated under endoplasmic reticulum stress, which promotes the apoptosis of cancer cells by negatively regulating ubiquitin-specific peptidase 39 (17). In gastric and breast cancers, miR-1281 also suppresses the malignant biological behaviors of cancer cells (18,31). In muscle-invasive bladder cancer, the expression of miR-1281 is also reduced (32). In the current study, it was observed that miR-1281 is a target of LINC00963, miR-1281 expression was downregulated in CRC tissues and cells, and the expression of miR-1281 was negatively correlated with LINC00963 expression in CRC tissues. Also, miR-1281 overexpression inhibited the proliferation, colony formation, migration and invasion of CRC cells, suggesting that miR-1281 is also a suppressor in CRC.

LncRNAs can function as competitive endogenous (ce)RNAs to modulate miRNA expression levels (33). According to previous reports, LINC00963 expression is upregulated in melanoma, and it promotes melanoma progression by sponging miR-608 and upregulating nucleus accumbens-associated protein 1 expression (34). In osteosarcoma, LINC00963 accelerates the growth and invasion of cancer cells by inhibiting miR-204-3p expression and inducing the expression of FN1 (26). In prostate cancer, LINC00963 promotes the expression of NOP2 by sponging the tumor suppressor miR-542-3p and facilitates the metastasis of prostate cancer (35). In breast cancer, LINC00963 promotes tumorigenesis and radioresistance by adsorbing miR-324-3p and inducing activated CDC42 kinase 1 expression (36). Additionally, LINC00963 can promote the progression of breast cancer by targeting miR-625 and upregulating high mobility group AT-hook 1 expression (37). In the present study, LINC00963 was identified as a molecular sponge for miR-1281, and TRIM65 was validated as one of the downstream targets of miR-1281. As a ubiquitin ligase, TRIM65 is reported to exert oncogenic functions in cancer biology. For example, TRIM65 negatively regulates the tumor suppressor p53 via ubiquitination in lung cancer cells, and silencing of TRIM65 inhibits the malignant phenotypes of lung cancer cells (38,39). In hepatocellular carcinoma, TRIM65 activates β-catenin signaling via the ubiquitylation of Axin1 (40). Notably, TRIM65 expression is upregulated in CRC, and TRIM65 targets Rho GTPase activating protein 35 to cause protein degradation, as well as promoting the proliferation, migration and invasion of CRC cells (41). A recent study reported that in glioma, TRIM65 is a target gene of miR-1281 (42). In the present study, LINC00963 was demonstrated to positively regulate TRIM65 expression by repressing miR-1281 expression. miR-1281 overexpression reversed the effects of LINC00963 overexpression on the phenotypes of CRC cells, which may be achieved by regulating the expression of TRIM65. Therefore, the present results proposed a ceRNA network consisting of LINC00963, miR-1281 and TRIM65, which was involved in CRC progression.

In conclusion, it was demonstrated that LINC00963 expression was upregulated and miR-1281 expression was downregulated in CRC. Also, knockdown of LINC00963 or overexpression of miR-1281 inhibited the proliferation, migration and invasion of CRC cells, while overexpressing LINC00963 or inhibiting miR-1281 induced the opposite effects. Additionally, LINC00963 can upregulate TRIM65 expression by sponging miR-1281. The present results offer useful indications for the diagnosis and treatment of CRC. However, in subsequent work, *in vivo* experiments are needed to further verify the current results.

## Supplementary Material

Supporting Data

## Figures and Tables

**Table I.** Characteristics of LINC00963 expression in patients with colorectal cancer.

		Expression of LINC000963		
			
Clinicopathological characteristics	Total (n=53)	High (n=27)	Low (n=26)	P-value
Sex				0.335
Male	26	15	11	
Female	27	12	15	
Age, years				0.907
<60	22	11	11	
≥60	31	16	15	
Location				0.132
Colon	30	18	12	
Rectum	23	9	14	
Tumor size, cm				0.039^a^
<5	23	8	15	
≥5	30	19	11	
Lymph node metastasis				0.020^a^
N0	26	9	17	
N1-N2	27	18	9	
Differentiation				0.171
Well	18	12	6	
Moderate	11	6	5	
Poor	24	9	15	
TNM stage				0.131
I–II	23	9	14	
III–IV	30	18	12	
Distant metastasis				0.464
No	21	12	9	
Yes	32	15	17	

a P<0.05.

## Data Availability

The datasets used and/or analyzed during the current study are available from the corresponding author on reasonable request.

## References

[1] Jiang X, Li Q, Zhang S, Song C, Zheng P (2019). Long noncoding RNA GIHCG induces cancer progression and chemoresistance and indicates poor prognosis in colorectal cancer. Onco Targets Ther.

[2] Liu L, Xie D, Xie H, Huang W, Zhang J, Jin W, Jiang W, Xie D (2019). ARHGAP10 inhibits the proliferation and metastasis of CRC cells via blocking the activity of RhoA/AKT signaling pathway. Onco Targets Ther.

[3] Nguyen LH, Goel A, Chung DC (2020). Pathways of colorectal carcinogenesis. Gastroenterology.

[4] Peng W, He D, Shan B, Wang J, Shi W, Zhao W, Peng Z, Luo Q, Duan M, Li B (2019). LINC81507 act as a competing endogenous RNA of miR-199b-5p to facilitate NSCLC proliferation and metastasis via regulating the CAV1/STAT3 pathway. Cell Death Dis.

[5] Abedini P, Fattahi A, Agah S, Talebi A, Beygi AH, Amini SM, Mirzaei A, Akbari A (2019). Expression analysis of circulating plasma long noncoding RNAs in colorectal cancer: The relevance of lncRNAs ATB and CCAT1 as potential clinical hallmarks. J Cell Physiol.

[6] Ye C, Shen Z, Wang B, Li Y, Li T, Yang Y, Jiang K, Ye Y, Wang S (2016). A novel long non-coding RNA lnc-GNAT1-1 is low expressed in colorectal cancer and acts as a tumor suppressor through regulating RKIP-NF-κB-Snail circuit. J Exp Clin Cancer Res.

[7] Huang L, Lin H, Kang L, Huang P, Huang J, Cai J, Xian Z, Zhu P, Huang M, Wang L (2019). Aberrant expression of long noncoding RNA SNHG15 correlates with liver metastasis and poor survival in colorectal cancer. J Cell Physiol.

[8] Sun X, Bai Y, Yang C, Hu S, Hou Z, Wang G (2019). Long noncoding RNA SNHG15 enhances the development of colorectal carcinoma via functioning as a ceRNA through miR-141/SIRT1/Wnt/β-catenin axis. Artif Cells Nanomed Biotechnol.

[9] Zheng K, Zhang TK (2020). LncRNA LINC00963 promotes proliferation and migration through the miR-124-3p/FZD4 pathway in colorectal cancer. Eur Rev Med Pharmacol Sci.

[10] Balacescu O, Sur D, Cainap C, Visan S, Cruceriu D, Manzat-Saplacan R, Muresan MS, Balacescu L, Lisencu C, Irimie A (2018). The impact of miRNA in colorectal cancer progression and its liver metastases. Int J Mol Sci.

[11] Lei K, Liang X, Gao Y, Xu B, Xu Y, Li Y, Tao Y, Shi W, Liu J (2017). Lnc-ATB contributes to gastric cancer growth through a MiR-141-3p/TGFβ2 feedback loop. Biochem Biophys Res Commun.

[12] Hannafon BN, Cai A, Calloway CL, Xu YF, Zhang R, Fung KM, Ding WQ (2019). miR-23b and miR-27b are oncogenic microRNAs in breast cancer: Evidence from a CRISPR/Cas9 deletion study. BMC Cancer.

[13] Yan L, You WQ, Sheng NQ, Gong JF, Hu LD, Tan GW, Chen HQ, Wang ZG (2018). A CREB1/miR-433 reciprocal feedback loop modulates proliferation and metastasis in colorectal cancer. Aging (Albany NY).

[14] Li P, Xue WJ, Feng Y, Mao QS (2015). MicroRNA-205 functions as a tumor suppressor in colorectal cancer by targeting cAMP responsive element binding protein 1 (CREB1). Am J Transl Res.

[15] Chen L, Gao H, Liang J, Qiao J, Duan J, Shi H, Zhen T, Li H, Zhang F, Zhu Z, Han A (2018). miR-203a-3p promotes colorectal cancer proliferation and migration by targeting PDE4D. Am J Cancer Res.

[16] Zhang H, Zhu M, Shan X, Zhou X, Wang T, Zhang J, Tao J, Cheng W, Chen G, Li J (2019). A panel of seven-miRNA signature in plasma as potential biomarker for colorectal cancer diagnosis. Gene.

[17] Jiang J, Ma B, Li X, Jin W, Han C, Wang L, Wang H (2018). miR-1281, a p53-responsive microRNA, impairs the survival of human osteosarcoma cells upon ER stress via targeting USP39. Am J Cancer Res.

[18] Liu G, Jiang Z, Qiao M, Wang F (2019). Lnc-GIHCG promotes cell proliferation and migration in gastric cancer through miR-1281 adsorption. Mol Genet Genomic Med.

[19] Yan S, Han B, Gao S, Wang X, Wang Z, Wang F, Zhang J, Xu D, Sun B (2017). Exosome-encapsulated microRNAs as circulating biomarkers for colorectal cancer. Oncotarget.

[20] Bertero L, Massa F, Metovic J, Zanetti R, Castellano I, Ricardi U, Papotti M, Cassoni P (2018). Eighth edition of the UICC classification of malignant tumours: An overview of the changes in the pathological TNM classification criteria-what has changed and why?. Virchows Arch.

[21] Amin MB, Greene FL, Edge SB, Compton CC, Gershenwald JE, Brookland RK, Meyer L, Gress DM, Byrd DR, Winchester DP (2017). The eighth edition AJCC cancer staging manual: Continuing to build a bridge from a population-based to a more ‘personalized’ approach to cancer staging. CA Cancer J Clin.

[22] Livak KJ, Schmittgen TD (2001). Analysis of relative gene expression data using real-time quantitative PCR and the 2(-Delta Delta C(T)) method. Methods.

[23] Paraskevopoulou MD, Vlachos IS, Karagkouni D, Georgakilas G, Kanellos I, Vergoulis T, Zagganas K, Tsanakas P, Floros E, Dalamagas T, Hatzigeorgiou AG (2016). DIANA-LncBase v2: Indexing microRNA targets on non-coding transcripts. Nucleic Acids Res.

[24] Agarwal V, Bell GW, Nam JW, Bartel DP (2015). Predicting effective microRNA target sites in mammalian mRNAs. Elife.

[25] Peng WX, Koirala P, Mo YY (2017). LncRNA-mediated regulation of cell signaling in cancer. Oncogene.

[26] Zhou Y, Yin L, Li H, Liu LH, Xiao T (2019). The LncRNA LINC00963 facilitates osteosarcoma proliferation and invasion by suppressing miR-204-3p/FN1 axis. Cancer Biol Ther.

[27] Wu JH, Tian XY, An QM, Guan XY, Hao CY (2018). LINC00963 promotes hepatocellular carcinoma progression by activating PI3K/AKT pathway. Eur Rev Med Pharmacol Sci.

[28] Lee SP, Hsieh PL, Fang CY, Chu PM, Liao YW, Yu CH, Yu CC, Tsai LL (2020). LINC00963 promotes cancer stemness, metastasis, and drug resistance in head and neck carcinomas via ABCB5 regulation. Cancers (Basel).

[29] Wang L, Han S, Jin G, Zhou X, Li M, Ying X, Wang L, Wu H, Zhu Q (2014). Linc00963: A novel, long non-coding RNA involved in the transition of prostate cancer from androgen-dependence to androgen-independence. Int J Oncol.

[30] Ganju A, Khan S, Hafeez BB, Behrman SW, Yallapu MM, Chauhan SC, Jaggi M (2017). miRNA nanotherapeutics for cancer. Drug Discov Today.

[31] Fan LY, Shi KY, Xu D, Ren LP, Yang P, Zhang L, Wang F, Shao GL (2019). LncRNA GIHCG regulates microRNA-1281 and promotes malignant progression of breast cancer. Eur Rev Med Pharmacol Sci.

[32] Pignot G, Cizeron-Clairac G, Vacher S, Susini A, Tozlu S, Vieillefond A, Zerbib M, Lidereau R, Debre B, Amsellem-Ouazana D, Bieche I (2013). microRNA expression profile in a large series of bladder tumors: Identification of a 3-miRNA signature associated with aggressiveness of muscle-invasive bladder cancer. Int J Cancer.

[33] Wang H, Wang G, Gao Y, Zhao C, Li X, Zhang F, Jiang C, Wu B (2018). Lnc-SNHG1 activates the TGFBR2/SMAD3 and RAB11A/Wnt/β-catenin pathway by sponging MiR-302/372/373/520 in invasive pituitary tumors. Cell Physiol Biochem.

[34] Jiao H, Jiang S, Wang H, Li Y, Zhang W (2018). Upregulation of LINC00963 facilitates melanoma progression through miR-608/NACC1 pathway and predicts poor prognosis. Biochem Biophys Res Commun.

[35] Sun F, Wu K, Yao Z, Mu X, Zheng Z, Sun M, Wang Y, Liu Z, Zhu Y (2020). Long noncoding RNA LINC00963 induces NOP2 expression by sponging tumor suppressor miR-542-3p to promote metastasis in prostate cancer. Aging (Albany NY).

[36] Zhang N, Zeng X, Sun C, Guo H, Wang T, Wei L, Zhang Y, Zhao J, Ma X (2019). LncRNA LINC00963 promotes tumorigenesis and radioresistance in breast cancer by sponging miR-324-3p and inducing ACK1 expression. Mol Ther Nucleic Acids.

[37] Wu Z, Wang W, Wang Y, Wang X, Sun S, Yao Y, Zhang Y, Ren Z (2020). Long noncoding RNA LINC00963 promotes breast cancer progression by functioning as a molecular sponge for microRNA-625 and thereby upregulating HMGA1. Cell Cycle.

[38] Li Y, Ma C, Zhou T, Liu Y, Sun L, Yu Z (2016). TRIM65 negatively regulates p53 through ubiquitination. Biochem Biophys Res Commun.

[39] Wang XL, Shi WP, Shi HC, Lu SC, Wang K, Sun C, He JS, Jin WG, Lv XX, Zou H, Shu YS (2016). Knockdown of TRIM65 inhibits lung cancer cell proliferation, migration and invasion: A therapeutic target in human lung cancer. Oncotarget.

[40] Yang YF, Zhang MF, Tian QH, Zhang CZ (2017). TRIM65 triggers β-catenin signaling via ubiquitylation of Axin1 to promote hepatocellular carcinoma. J Cell Sci.

[41] Chen D, Li Y, Zhang X, Wu H, Wang Q, Cai J, Cui Y, Liu H, Lan P, Wang J (2019). Ubiquitin ligase TRIM65 promotes colorectal cancer metastasis by targeting ARHGAP35 for protein degradation. Oncogene.

[42] Hu G, Liu N, Wang H, Wang Y, Guo Z (2019). LncRNA LINC01857 promotes growth, migration, and invasion of glioma by modulating miR-1281/TRIM65 axis. J Cell Physiol.

